# Orientation of Mitotic Spindles during the 8- to 16-Cell Stage Transition in Mouse Embryos

**DOI:** 10.1371/journal.pone.0008171

**Published:** 2009-12-04

**Authors:** Nicolas Dard, Sophie Louvet-Vallée, Bernard Maro

**Affiliations:** 1 CNRS, UMR7622 - Laboratoire de Biologie Cellulaire du Développement, Paris, France; 2 UPMC Univ. Paris 06, UMR7622 - Laboratoire de Biologie Cellulaire du Développement, Paris, France; 3 CNRS, Paris, France; 4 Sackler Faculty of Medicine, Tel Aviv University, Ramat Aviv, Israel; CNRS UMR6543 - Université de Nice - Sophia Antipolis, France

## Abstract

**Background:**

Asymmetric cell divisions are involved in the divergence of the first two lineages of the pre-implantation mouse embryo. They first take place after cell polarization (during compaction) at the 8-cell stage. It is thought that, in contrast to many species, spindle orientation is random, although there is no direct evidence for this.

**Methodology/Principal Findings:**

Tubulin-GFP and live imaging with a spinning disk confocal microscope were used to directly study spindle orientation in whole embryos undergoing the 8- to 16-cell stage transition. This approach allowed us to determine that there is no predetermined cleavage pattern in 8-cell compacted mouse embryos and that mitotic spindle orientation in live embryo is only modulated by the extent of cell rounding up during mitosis.

**Conclusions:**

These results clearly demonstrate that spindle orientation is not controlled at the 8- to 16-cell transition, but influenced by cell bulging during mitosis, thus reinforcing the idea that pre-implantation development is highly regulative and not pre-patterned.

## Introduction

During development, asymmetric cell divisions, leading to the formation of two different daughter cells, is one of the major mechanisms involved in the generation of cell diversity. Prior to cell division, the cell has to be polarized in order to allow an asymmetric division. Then, cellular components can be segregated differentially in the two daughter cells if the mitotic spindle aligns with the axis of polarity. In many embryos, specific mechanisms are involved in the control of mitotic spindle orientation. For example, in *C.elegans*, just after fertilization, the two pronuclei and associated centrosomes are positioned in the posterior half of the zygote with the centrosomes aligned perpendicular to the anterior-posterior axis. Then, the two pronuclei migrate to the centre of the zygote and rotate 90°. During anaphase, the mitotic spindle moves toward the posterior pole resulting in asymmetric cell division. The orientation and positioning of the spindle require the anchorage of astral microtubules to the cortex and a conserved set of polarity regulators, the partitioning defective complex (Par complex) [1]. In ascidian embryos at the 8-cell stage, the two posterior blastomeres undergo a series of asymmetric divisions that separate muscle cell precursors from germline ones. These divisions are directed by a macroscopic cortical structure, the centrosomes attracting body (CAB), which controls spindle positioning and distribution of mRNA determinants. Proteins of the Par complex accumulate in the CAB at the onset of asymmetric divisions [2]. These studies highlight the major role of PAR complex and centrosome in spindle orientation.

The asymmetric cell divisions observed during pre-implantation development of the mouse embryo differ from these models since centrosomes are absent until the blastocyst stage [3]. Two distinct cell populations are first observed at the 16-cell stage that can be distinguished by both their position (outside and inside) and their phenotype (polarized and non-polarized, respectively). These two cell types derive from 8-cell blastomeres that polarize at compaction along a radial axis, allowing asymmetric cell divisions to take place. Whether or not a blastomere divides asymmetrically does not seem to be determined randomly since early dividing blastomeres tend to do so more frequently, contributing more cells to the inner cell mass lineage [4]-[6]. However, the orientation of the spindle does not seem to be tightly controlled since there is a great variability in the number of inner cells at the 16-cell stage [7]-[9]. Moreover, experiments performed on isolated 8-cell blastomeres, cultivated either as singleton or in pairs, where cleavage planes were observed under the dissecting microscope, demonstrated that these blastomeres could divide either symmetrically or asymmetrically [10]. These observations led to the conclusion that spindle orientation was random in 8-cell blastomeres. However, using lineage marker analysis and isolated pairs of 16-cell blastomeres, it was shown that cell shape was able to influence spindle orientation at the 16- to 32-cell transition [10], [11].

Although a recent study suggests that the pattern of symmetric and asymmetric cell divisions might not be random [12], a direct evidence for a predetermined orientation of spindles at the 8- to 16-cell stage has yet to be demonstrated. In this paper, we used tubulin-GFP and live imaging using a spinning disk confocal microscope to directly study spindle orientation in whole embryos achieving the 8- to 16-cell stage transition. The methodology used is non invasive and non deleterious since the embryos reached the blastocyst stage. This approach allowed us to determine that there is no predetermined cleavage pattern in 8-cell compacted mouse embryos and that mitotic spindle orientation in live embryo is only modulated by the extent of cell rounding up during mitosis.

## Materials and Methods

### Ethic Statement

All experiments performed in the present study were approved by the French Agriculture Department (agreement #A75-05-13). All animals used in experiments reported in this publication were housed and handled by persons skilled by institutional committee according to CNRS and French Agriculture Department.

### Recovery and Culture of Mouse Embryos

Recovery and culture of embryos were performed as described previously [13]. Briefly, 9 to 12 weeks old females OF1 (Charles River) were superovulated by intraperitoneal injection of 5 UI Pregnant Mare Serum gonadotrophin (PMS, Intervet) and 5 UI human Chorionic Gonadotrophin (hCG, Intervet), 48 hours later. Females were mated with OF1 males (fertilization occurs about 12 hours post-hCG). Two-cell stage embryos were collected by flushing oviducts in M2+BSA (4mg/ml) medium and then cultured in T6+BSA under paraffin oil at 37°C in 5% CO_2_.

### Plasmids, Synthesis of mRNA, and Microinjection

β5-tubulin-GFP (gift of B. Ludin) was cloned into pRN3 plasmid. In vitro synthesis of mRNA was performed as described previously [14]. Microinjection of synthetic mRNA was performed into the cytoplasm of the two cells of 2-cell stage embryos (35-38 hrs post-fertilization) as described previously [14].

### Time-Lapse Microscopy

Embryos were cultured in T6+BSA under paraffin oil in a specially designed chamber adapted to the inverted microscope (Axiovert M200, Zeiss), maintained at 38°C, in an atmosphere of 96% air with 4% CO2. The microscope was equipped with a spinning disk (Yokogawa CSU-10) and an EMCCD camera (Hamamatsu). The system was driven by the Volocity Acquisition software (Improvision – Perkin Elmer) running on a Mac Pro (Apple Computer). Series of confocal images (z = 1.5 µm) were recorded every 20 min for each channel used (transmission and green fluorescence). In these conditions, embryos develop to the blastocyst stage.

### Determination of Angles and Measurement of Cell Bulging during Mitosis

**Figure 1:**
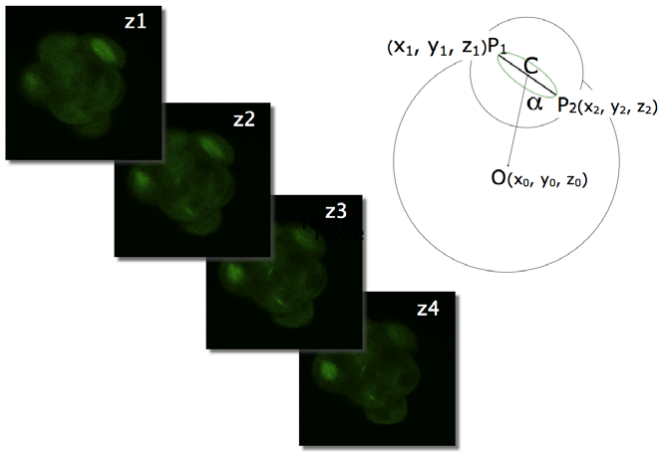
Determination of spindle poles and embryo centre coordinates. For a given spindle, the position of three points were determined, by moving through the stack of images (z1, z2, z3, …): the two poles of the spindle (P1 and P2) and the centroid of the embryo (O). The position of the centre of the spindle (C) and the value of the α angle were calculated using the coordinates of these 3 points.

Using the Volocity Visualization/Quantitation software package (Improvision – Perkin Elmer) running on a Mac Pro (Apple Computer), the coordinates (x, y, z) of the two poles of the mitotic spindle (in all cells of the embryo) and of the centroid of the embryo were determined (Fig. 1). Then, the angle between the vector determined by the two spindle poles (P_1_P_2_) and the vector going from the centroid of the embryo to the middle of the spindle (OC) was calculated using the iWorks Numbers software (Apple Computer). To estimate the extent of bulge of the mitotic cells, we selected the view passing through the spindle and displaying the largest perimeter. Then the distance between the two points of contact of the bulge with the embryo (d) and the length of the two segments (h, H) corresponding to the bisecting line of d were measured. The surface of S_1_ (corresponding to half of an ellipsoid) and S_2_ (corresponding to a truncated circle) was calculated using the Numbers software. Statistical analysis was performed using the Prism and InStat software packages (GraphPad).

### Quantification of the Number of Inside Cells at the 16-Cell Stage

16-cell embryos were fixed in 3.7% formaldehyde (BDH) in PBS for 30 minutes at 37°C, and neutralized with 50 mM NH_4_Cl in PBS for 10 minutes. Samples were then post-permeabilized with 0.25% Triton X-100 in PBS for 10 minutes. Actin staining was performed by a 15 minutes incubation of embryos with 1 µg/mL TRITC-conjugated phalloïdin (Sigma) at room temperature. Hoescht was used to stain chromatin. Samples were mounted in citifluor and observed under a Zeiss Axiovert M200 inverted microscope equipped with a spinning-disk confocal system. For each blastomere we checked on serial sections whether part of its cortical domain was exposed at the surface of the embryo.

## Results and Discussion

**Figure 2:**
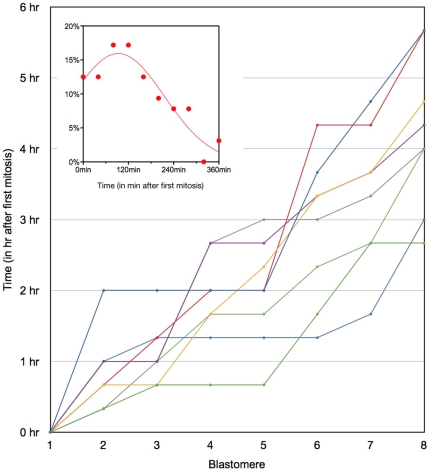
Timing of the eight mitotic divisions in 8-cell stage mouse embryos. Each colour corresponds to a given embryo. Timing of metaphase (Y axis) was used for each blastomere (X axis). Inset: distribution of the timing of metaphase in the population of embryos studied (the Y axis corresponds to the percentage of blastomeres dividing at a given time).

2-cell stage mouse embryos were injected in both blastomeres with a tubulin-GFP mRNA. They were then cultured in a specially designed chamber and imaged with a spinning-disk confocal microscope. Whole embryos were scanned along the z axis and series of confocal images (z = 1.5 µm) were recorded every 20 min for each channel used (transmission and green fluorescence) during 20 hours. Metaphase was used as a reference to determine the timing of mitosis (one image before anaphase). Metaphase was also used to measure spindle size and orientation (Fig. 1). We must point out that once the spindle formed in prometaphase, its orientation did not change during the period of prometaphase to telophase. The time when the first 8-cell blastomere divided was used as time 0 for a given embryo (Fig. 2). For these studies, we used only embryos where all 8 mitotic spindles could be observed and the time required for the transition from the 8-cell stage (first division) to the 16-cell stage (eighth division) was not longer than 6 hours. According to these restrictive criteria, 8 embryos (out of a 20 recorded in 4 different experiments) – corresponding to 64 blastomeres – were further analyzed (5 embryos took longer than 6 hours to divide and we were not able to observe all 8 spindles in the other embryos).

### Timing of Divisions

As observed previously, mitotic divisions at the 8- to 16- cell stage transitions are asynchronous (Fig. 2). Moreover the distribution of the timing of mitosis for all embryos passed the D'Agostino & Pearson «omnibus K2» normality test (Fig. 2, inset) showing that these data are consistent with a Gaussian distribution (Gaussian goodness of fit: r^2^ = 0.8870). This distribution implies that the later the cell divides, the more asynchronous it is: 60% of the cells divide during the first 2 hours and 40% during the last 4 hours. Moreover, the timing of the 8 divisions within an embryo differs greatly from embryo to embryo (Fig. 2).

### Spindle Orientation Distribution

**Figure 3:**
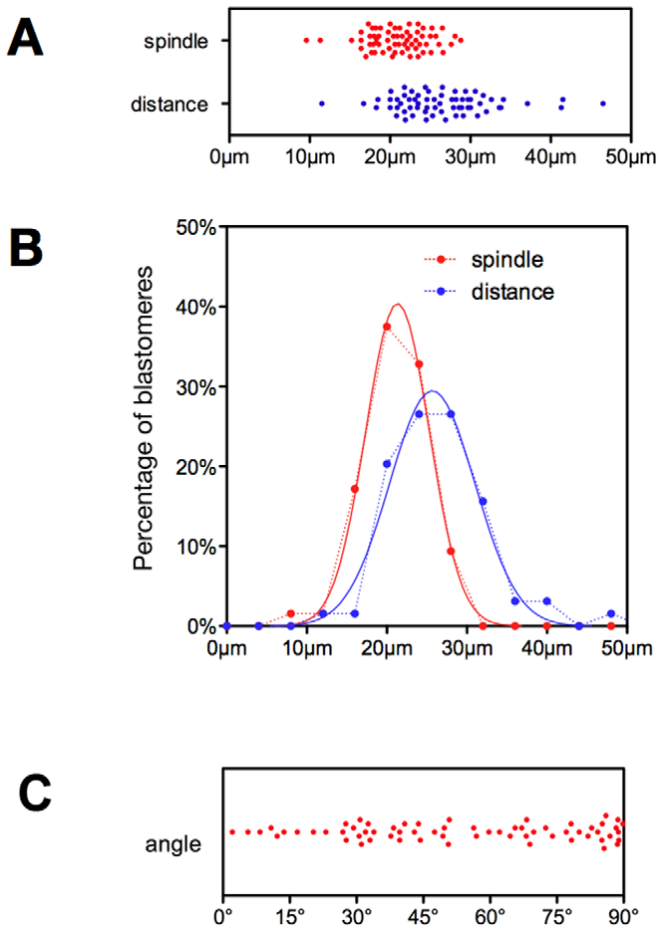
Dispersed distribution of the angle between the spindle and the radial axis. Distributions of the spindle size (A-B, red), distance from the centroid (A-B, blue) and α angle value (C). In B, the same populations (spindle size and distance from the centroid) were plotted as a frequency distribution (every 4 µm). The dashed lines correspond to our data and the plain lines to the curve fits.

The size of the metaphase spindles (Fig. 3A), was 21.0±3.7 µm (mean±SD) in all embryos (Gaussian goodness of fit: r^2^ = 0.9976; Fig. 3B), 70% of the population measuring between 17.5 and 27.5 µm. There was no difference in spindle length between early (first three) and late (last three) dividers: 20.6±4.0 µm versus 19.6±3.8 µm (p = 0.3793 using the unpaired t-test). The distance from the centroid of the embryo to the centre of the spindle (Fig. 3A, B) was slightly more variable: 26.5±6.0 µm (Gaussian goodness of fit: r^2^ = 0.9747).

**Figure 4:**
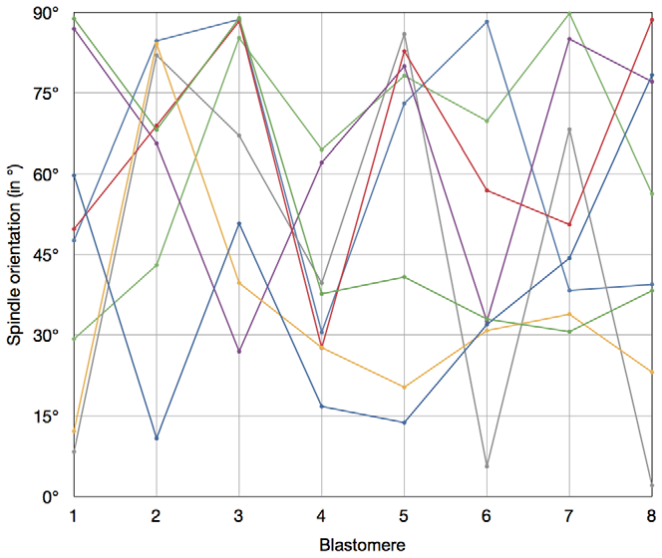
Orientation of the eight mitotic spindles in 8-cell stage mouse embryos. Each colour corresponds to a given embryo. Spindle orientation (in degrees; Y axis) was measured for each blastomere (X axis). Blastomeres were ranked according to the timing of mitosis. No define pattern could be observed.

**Figure 5:**
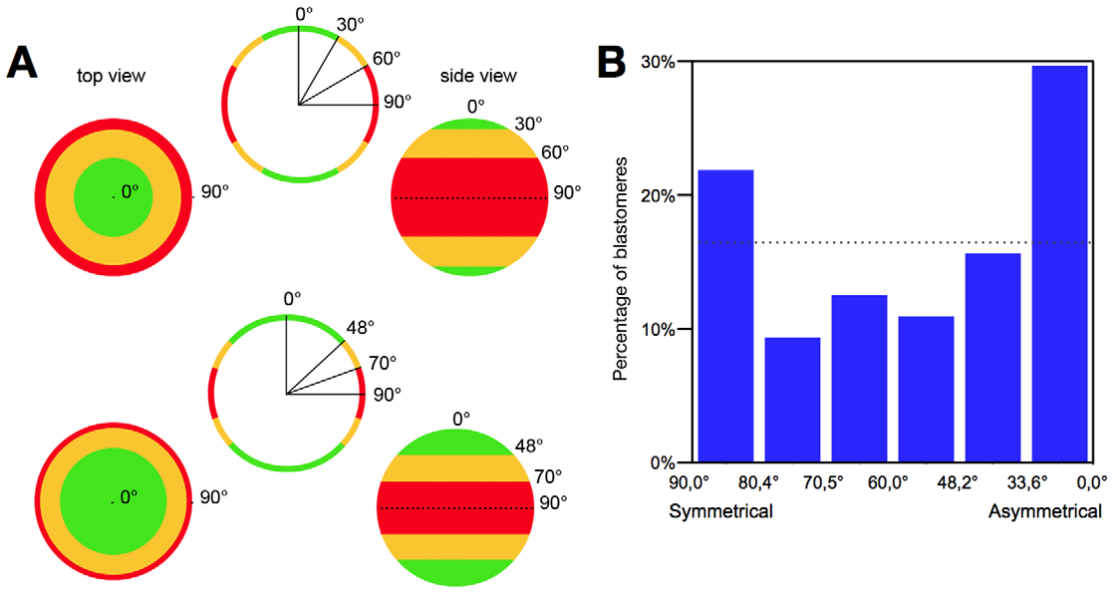
Spindle orientation in 8-cell stage embryos. A: Schematic representation of the probability of spindle orientation distribution. Since the spindle can orient in a 3D space, and not on a 2D plane, the probability for the spindle to be in a given range of angles is proportional to a «stripe» of the surface of a sphere. Therefore, this probability is proportional to cosine (α) rather than to α itself. This is illustrated on these three colours balloons, viewed from the top and the side. If the ranges are proportional to α, then the surfaces covered by each of the three colours are different (top). When the ranges are proportional to cosine (α), each colour covers the same area of the surface (bottom). B: Distribution of α according to cosine (α): the cosine of the angles (X axis) shown corresponds to multiple of 0.166 (since cosine (α) varies between 0 and 1). The Y axis corresponds to the percentage of spindle oriented with a given angle. A non-random distribution is observed, with an increase for the two extreme ranges suggesting that spindle orientation is not completely random. The dash line corresponds to the expected percentage for each “α” angle value if the spindle orientation was random.

In contrast, the distribution of the α angle (corresponding to spindle orientation) was very dispersed (Fig. 3C), ranging from 2.1° to 89.8°, with a median at 50.7°. Again, when the orientation of the 8 spindles of a given embryo was compared with those of other embryos, no define pattern could be observed (Fig. 4). The number of blastomeres with α>60° was much greater than the one with α<30° (Fig. 3C). This may suggest that this distribution is not random. However, the probability for the spindle to be in a given range of angles is proportional to a «stripe» of the surface of a sphere (Fig. 5A top), not to an arc at the periphery of a circle (Fig. 5A bottom) since the spindle can take any orientation in a 3D space and is not limited to a 2D plane. Thus, this probability is proportional to cosine (α) rather than to α (as it is in a 2D plane). When we sliced the distribution of α according to cosine (α), we observed a non-random distribution (Fig. 5B), with an increase for the two extreme ranges (80°-90° and 34°-0°) suggesting that spindle orientation is not completely random.

### Spindle Orientation and Asymmetric Divisions

**Figure 6:**
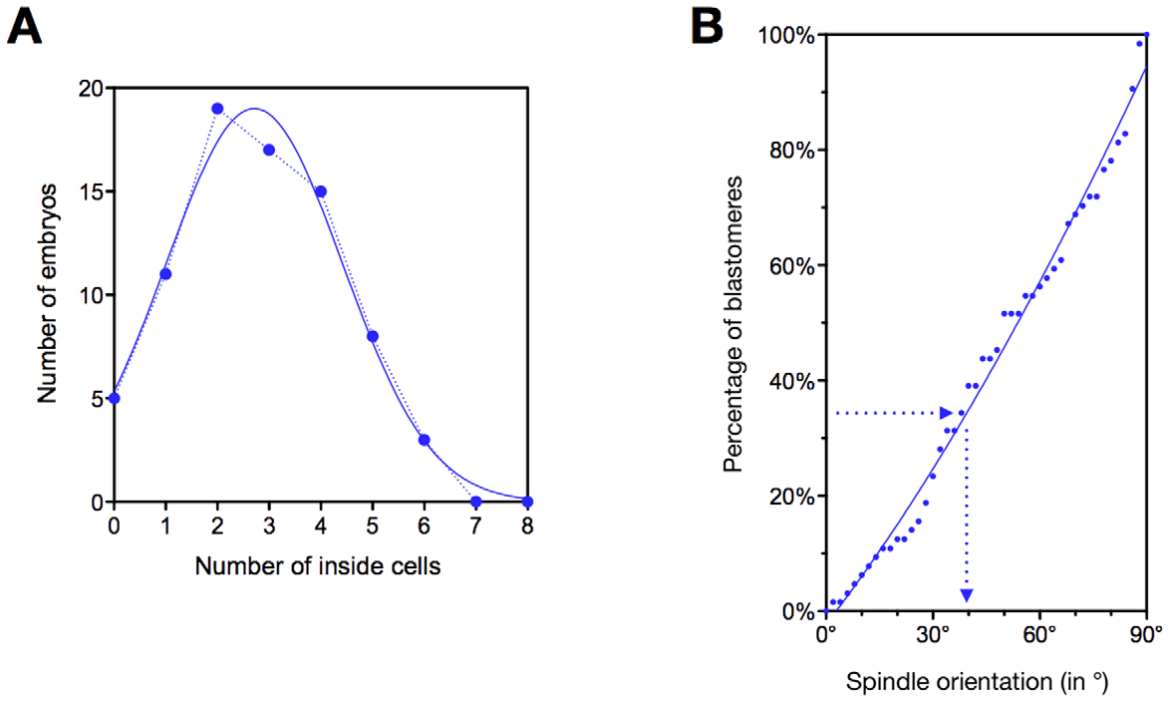
Spindle orientation and asymmetric divisions. A: The number of inside cells was measured in a population of 78 fixed 16-cell stage embryos. The dotted line corresponds to the experimental values and the plain line to the best fit. The mean number of inside cells was 2.8, corresponding to 35% of asymmetric divisions. B: Cumulative distribution of the α angle in living blastomeres (n = 64). Blastomeres were sorted according to the value of the α angle. Each point on the graph corresponds to an increment of 2°. The X axis corresponds to the α angle. The Y axis corresponds to the percentage of the population with an α angle smaller or equal to a given value. The best fit line was plotted using the least square method. 35% of asymmetric divisions correspond to a threshold angle of about 40° (arrows).

Since spindle orientation controls asymmetric divisions, we attempted to determine the threshold angle required for the blastomere to divide asymmetrically or symmetrically using an indirect approach. The number of inside cells (corresponding to the number of asymmetric divisions during the previous mitosis) was measured in a population of 78 fixed 16-cell stage embryos stained for chromatin and actin to allow an easy screening of cell periphery. The mean number of inside cells was 2.8 cells (Fig. 6A) corresponding to 35% of asymmetric divisions (2.8 asymmetric divisions /8 total number of divisions per embryo). In our sample population, this corresponds to a threshold angle of about 40° (Fig. 6B), suggesting that blastomeres with a spindle oriented in the range between 0° and 40° divide asymmetrically, while those with a spindle oriented in the range between 40° and 90° divide symmetrically.

### Blastomere Bulging during Mitosis Influences Spindle Orientation

**Figure 7:**
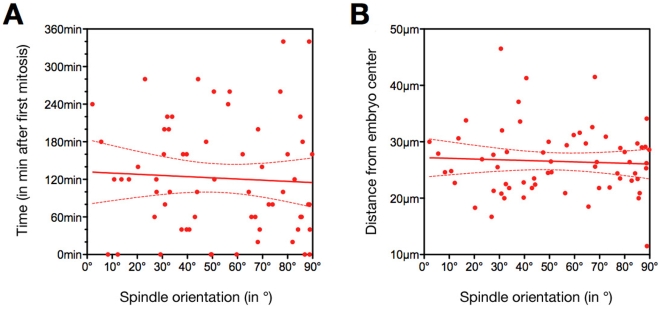
Spindle orientation is not modulated by the timing of division nor the position of blastomeres. Correlation between spindle orientation (X axis) and the timing of division (A; Y axis) or the distance from the embryo centre (B; Y axis).

In order to look for factors that could influence spindle orientation, we checked whether the timing of division (Fig. 7A) or the distance from the centre of the spindle to the embryo centre (Fig. 7B) could be involved. In both case we did not observe any correlation (P = 0.7522 for timing and P = 0.7944 for the distance from the centre). Therefore, neither the timing of division nor the distance from the embryo centre influences spindle orientation.

**Figure 8:**
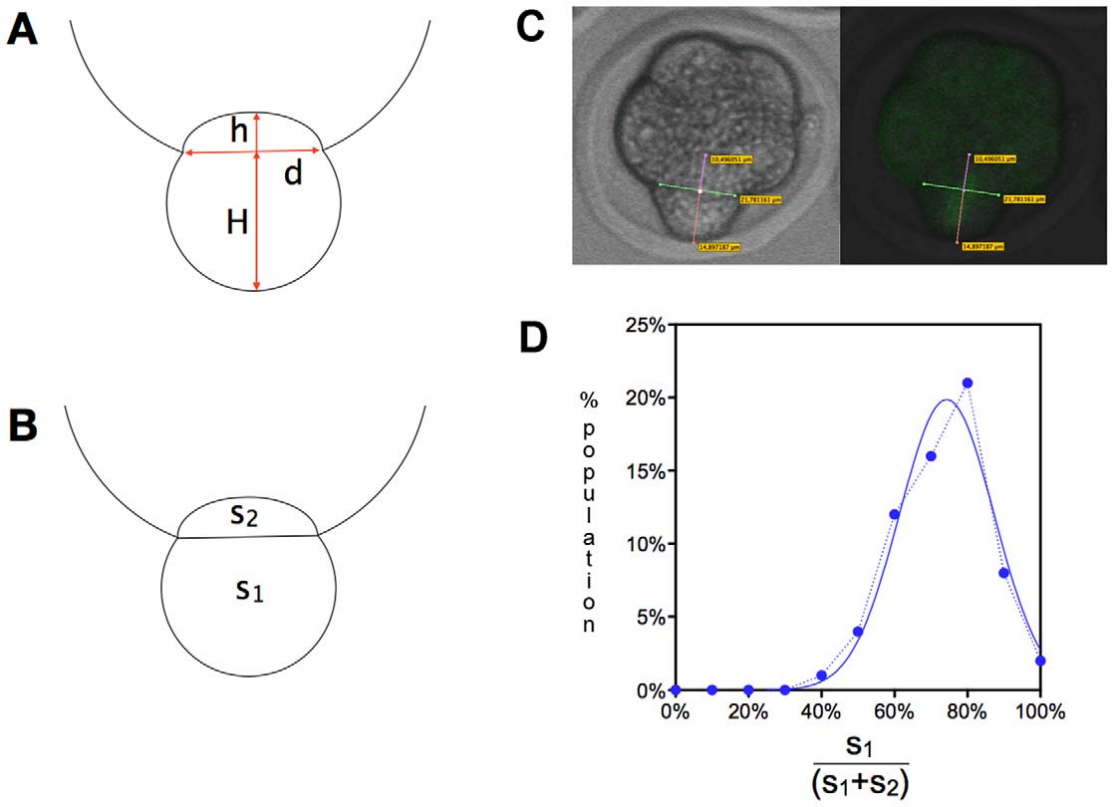
Measurement of blastomere bulging during mitosis. A-C: The measurements (A) required to estimate blastomere bulging were performed on image stacks (C). The surface of S_1_ (corresponding to half of an ellipsoid) and S_2_ (corresponding to a truncated circle) was then calculated (B). An example is shown in C where both fluorescence and transmitted light images were used. D: Distribution of the «bulging» index. The Y axis corresponds to the percentage of blastomeres with a given bulging index (X axis).

During mitosis, blastomeres round up and bulge out off the compacted embryo. To quantify this effect, we made use of the ability of observing this effect in 3D in our image stacks and measured the extent of bulging (Fig. 8A-C). Using these measurements, we calculated a «bulge» index corresponding to the ratio of the surface bulging out (S_1_) on the total surface of the blastomere (S_1_+S_2_) (Fig. 8D). This index varied from 40% to 99% with a mean value of 72%±13% (mean±SD). The distribution of this «bulge» index (Fig. 8D) passed the D'Agostino & Pearson «omnibus K2» normality test showing that these data are consistent with a Gaussian distribution (Gaussian goodness of fit: r^2^ = 0.9611).

**Figure 9:**
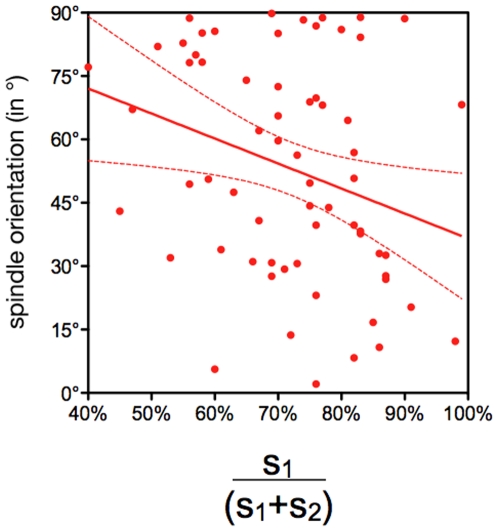
Blastomere bulging during mitosis influences spindle orientation. Correlation between spindle orientation (Y axis) and the blastomere bulging index (S1/(S1+S2); X axis).

**Figure 10:**
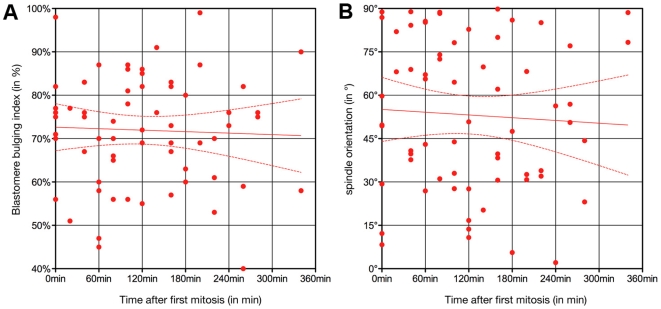
Spindle orientation and bulging in early dividing blastomeres. Neither blastomere bulging during mitosis (A) nor spindle orientation (B) is influenced in early dividers. Each point corresponds to a blastomere.

We observed a significant correlation (P = 0.0204) between the «bulge» index and spindle orientation (Fig. 9). The higher the «bulge» index, the lower the angle is. This suggests that blastomeres that pop out almost completely off the embryo divide more frequently asymmetrically, while those with a large part remaining within the embryo will divide more symmetrically. In contrast, there was no correlation between the timing of division and cell bulging during mitosis (Fig. 10A) or spindle orientation (Fig. 10B). However we observed that only 25% of early dividers (first three divisions; Fig. 4) had a spindle oriented between 0° and 40° while 54% of late dividers (last three divisions; Fig. 4) were in this case (although the distribution of the two populations is not significantly different P = 0.2211).

### Conclusion

Using videomicroscopy we observed for the first time the orientation of mitotic spindles during the 8- to 16-cell stage transition in living embryos. This approach allowed us to determine that there is no predetermined cleavage pattern in 8-cell compacted mouse embryos and that mitotic spindle orientation in live embryo is random, being only modulated by the extent of cell rounding up during mitosis. Recently, another study looked at cleavages in preimplantation mouse embryos [12]. In this paper, the orientation of the mitotic spindle was not monitored directly and the division type (asymmetric or symmetric) was inferred from the position of the daughter cells. However, a rapid relocation of some daughter cells with a change in phenotype can occur rapidly after mitosis [15]. Intercellular adhesion and cortical tension are major factors able to modulate bulging [15]. Zona pellucida also exerts a constraining effect on this event. Thus both intrinsic and extrinsic factors can modulate cell rounding up and thus spindle orientation. However, it was shown that early dividing blastomeres tend to divide asymmetrically more frequently, contributing more cells to the inner cell mass lineage [4]-[6]. This increase in asymmetric division might be controlled through the modulation of spindle orientation. From our data, this is clearly not the case: we observed that only 25% of early dividers had a spindle oriented between 0° and 40° while 54% of late dividers were in this case. This difference may be explained by the fact that the blastomeres were disaggregated and reaggregated in the other studies, which disturbs the timing of division.

Finally, our study suggests that spindle orientation, either directly or indirectly, is not used at the transition from the 8-cell to the 16-cell stage to modulate the number of asymmetric divisions. Another mechanism that could control the proportion of asymmetric divisions at the 8- to 16- cell transition is the size of the microvillus pole generated at the cell apex during the 8-cell stage (through intercellular contacts) and its inheritance during the next mitosis. This would influence the type of progeny (the smaller the pole, the greater the number of asymmetric divisions) [10]. However, this remains to be investigated.
